# Dihydrocoumarin Transcriptionally Activates *EcIAA9* to Repress Root Growth of *Echinochloa crus−galli*

**DOI:** 10.3390/biology15211989

**Published:** 2026-11-03

**Authors:** Xuan Chen, Jiadi Zhang, Jiali Chen, Di Wu, Nufen Li, Shangfeng Zhou, Lifeng Wang, Haona Yang

**Affiliations:** 1 Longping Agricultural College, Hunan University, Changsha 410128, China; cxuan5885@163.com (X.C.);; 2 Hunan Institute of Plant Protection, Hunan Academy of Agricultural Sciences, Changsha 410128, China; 3 College of Plant Protection, Hunan Agricultural University, Changsha 410128, China; 4 Yuelushan Laboratory, Changsha 410128, China

**Keywords:** dihydrocoumarin, allelochemical, *Echinochloa crus−galli*, *EcIAA9−EcARF7* module, auxin signaling

## Abstract

*Echinochloa crus−galli* is one of the world’s ten most pernicious weeds and is mainly controlled by chemical herbicides. However, the application of herbicides over long periods has escalated weed resistance, highlighting the need for eco−friendly alternatives. As a degradable natural allelochemical, dihydrocoumarin displays auxin−like activity and inhibits root growth in *E. crus−galli* in a manner dependent on concentration, phenotypically mimicking exogenous IAA. Previous studies have shown that dihydrocoumarin suppresses weed growth largely via auxin signaling. Here, we identified *EcIAA9*, a gene that responds to dihydrocoumarin and encodes a nuclear protein interacting with *EcARF7*. Heterologous rice assays showed that at 25 mg/L dihydrocoumarin, *EcIAA9* overexpression increased primary−root inhibition from 30.81% (WT) to 54.44% and 52.00% (*EcIAA9*−OE). Conversely, *OsIAA9* knockout lines consistently exhibited a milder root inhibition trend compared to the overexpression lines. These differential phenotypes collectively establish *IAA9* as a key positive regulator in this pathway. Notably, *EcIAA9* overexpression rice showed different responses to dihydrocoumarin and IAA, and combined treatments produced additive inhibitory effects. Knockout of *OsARF7* failed to fully abolish dihydrocoumarin−triggered inhibition, indicating that *ARF7* participates in this regulatory module, although functional compensation among ARF homologs may underlie this observation. In summary, the *EcIAA9–EcARF7* regulatory module contributes to the repression of root growth triggered by dihydrocoumarin. These findings show that dihydrocoumarin phenotypically mimicked the inhibitory effect of IAA on barnyardgrass but acted through a somewhat different mechanism. This study provides new insights into the crosstalk between allelochemicals and auxin signaling.

## Simple Summary

*Echinochloa crus−galli* is a major weed controlled by herbicides, but resistance is rising, so eco−friendly alternatives are needed. Dihydrocoumarin is a natural compound that inhibits barnyardgrass roots like the hormone auxin. We studied its molecular mechanism. We identified *EcIAA9*, a gene responding to dihydrocoumarin that interacts with *EcARF7*. In rice, overexpressing *EcIAA9* increased sensitivity to dihydrocoumarin, whereas knocking out its native counterpart resulted in a conspicuously milder inhibition compared to the overexpressors. These contrasting phenotypes collectively establish *IAA9* as a key positive regulator in this pathway. Dihydrocoumarin and auxin acted differently, with additive effects when combined. Removing *EcARF7*’s rice counterpart did not fully block inhibition, suggesting other proteins compensate. Together, *EcIAA9* and *EcARF7* suppress root growth. This shows a natural chemical mimics auxin but uses a different mechanism, offering clues for new eco−friendly weed killers.

## 1. Introduction

Weeds are among the most detrimental biotic stressors in agricultural systems, severely constrain high−yielding, high−quality crop production, causing potential yield losses of up to 34% in major crops, far exceeding losses from animal pests (18%) and plant pathogens (16%) [1], and can even lead to complete crop failure [2]. Considered one of the most troublesome weeds in rice fields, *Echinochloa crus−galli* infests and damages rice throughout the entire growing season, representing a primary biotic limitation to efficient rice production [3]. In tropical and subtropical areas, yield reductions caused by this weed can reach 50%, varying with infestation levels and environmental factors [4]. Since the 1990s, chemical herbicides have largely dominated weed management strategies due to their efficiency and cost efficiency, especially following the introduction of genetically modified crops that are resistant to herbicides [5]. However, their overuse has hastened the development of weeds resistant to herbicide [6,7]. To date, a total of 540 unique cases of resistance involving weed species and sites of action have been documented worldwide, involving 275 species, including 156 dicots and 119 monocots (https://www.weedscience.org). Among these resistant weeds, *E. crus−galli* deserves special attention, as it has rapidly evolved resistance to multiple herbicides, including cross resistance and multiple resistance [8,9]. This evolution erodes the efficacy of existing herbicides and weakens conventional control measures. The growing prevalence of resistance, coupled with restrictions on herbicides that pose high risks, underscores the urgent need for sustainable weed management alternatives. Consequently, the development and implementation of effective control strategies, such as bioherbicide application and the discovery of novel herbicide targets, are crucial for mitigating the impact of *E. crus−galli* on rice cropping systems [5,10,11]. In particular, strategies based on allelopathy, including the use of allelochemicals as bioherbicides, have attracted growing interest.

Allelopathy, defined as the chemically mediated interference between plants, has emerged as a crucial element of integrated weed management systems, particularly in sustainable and organic agriculture [12,13,14]. Utilizing allelopathic crops, plant residues or mulches, and bioherbicides formulated with allelochemicals can effectively suppress weeds and reduce reliance on synthetic herbicides [15]. These strategies may reduce reliance on synthetic herbicides and, in some systems, lower environmental risks; however, field−scale evidence, non−target risk assessment, and cost–benefit analysis are needed before environmental or economic benefits can be claimed [16,17].

Allelochemicals have been classified into 14 distinct chemical categories. At the physiological level, they disrupt cellular homeostasis by inducing reactive oxygen species (ROS) bursts, altering ion flux, and activating mitogen−activated protein kinase (MAPK) and leucine−rich repeat (LRR) receptor−like kinase signaling cascades [13,18]. These disruptions ultimately impair membrane permeability, water and ion uptake, photosynthesis, and respiration, phytohormone balance, as well as cell division and elongation [19,20,21,22,23]. At the molecular level, allelochemicals are known to regulate plant growth through complex genetic networks involving transcription factors and secondary metabolite biosynthesis pathways [24,25,26,27,28,29].

In our previous study, we found that the decomposed extract of *Malachium aquaticum* effectively inhibited *E. crus−galli* seed germination [3]. Further extraction and identification revealed that the primary allelochemical in the extract was dihydrocoumarin, which exhibited stronger inhibitory effects on *E. crus−galli* root growth than coumarin—a compound widely regarded as a representative allelochemical of the coumarin class. Dihydrocoumarin, a benzopyran derivative within this group [30], has been shown to interfere with auxin signal transduction in *E. crus−galli*, an effect closely associated with its inhibitory action on root growth [31].

Auxin regulates plant growth and development, and plays a crucial role in establishing the final architecture of the root system [32]. Over recent decades, the TIR1/AFB−Aux/IAA−ARF transcriptional pathway has been extensively characterized as the primary auxin signaling mechanism [33]. Composed of specific Aux/IAA and ARF combinations, these modules govern root architecture and adaptive growth, including primary root elongation, lateral root formation, and deep root penetration [34,35,36]. *Aux/IAA* genes encode primary transcriptional repressors and are among the earliest and most rapidly responsive to auxin [37]. Numerous reported *Aux/IAA* genes are involved in modulating root development and growth. In particular, overexpression of *Aux/IAA* genes disrupts root system architecture, causing abnormal tropic responses, reduced primary root elongation, and defective lateral root formation [38,39,40]. As key downstream targets of Aux/IAA repressors, ARF proteins participate in functional diversification to drive the initiation of distinct root types [41]. Auxin signaling dependent on Aux/IAA−ARF coordinately governs the development of distinct root structures including lateral roots and cortical aerenchyma, albeit with differential timing and regulatory patterns [42]. Importantly, environmental signals are capable of reshaping the outputs of this auxin signaling module. For example, high temperature facilitates root cell elongation by enhancing the transcriptional activity of *ARF7/19* even with increased Aux/IAA stability [43]. Water gradients further tune this cascade via SUMO modification of *ARF7*, which recruits IAA3 to suppress lateral root formation in root zones under water limitation [44]. In addition, phenolic allelochemicals such as coumarin interfere with Aux/IAA protein turnover and perturb root development dependent on auxin [45]. Against this background, we focused on *EcIAA9*, an Aux/IAA family gene showing the strongest transcriptional response in *E. crus−galli* upon dihydrocoumarin exposure.

To investigate its potential role, we integrated exogenous IAA treatment and compared the responses of *E. crus−galli* and heterologous transgenic rice to dihydrocoumarin and IAA. Given that dihydrocoumarin phenocopies auxin’s effects on root development, we further integrated exogenous IAA treatment to compare the biological responses of *E. crus−galli* and transgenic rice to dihydrocoumarin and IAA. We identified ARF proteins that physically interact with *EcIAA9* and systematically characterized the genetic responses of *EcIAA9* overexpressing, *OsIAA9* knockout, and *OsARF7* knockout lines to single and combined treatments of dihydrocoumarin and IAA. This study aimed to investigate the potential role of the *EcIAA9–ARF7* module in dihydrocoumarin−induced root growth repression and to compare the phenotypic responses to dihydrocoumarin and IAA; mechanistic differences remain to be established. These findings may help clarify how dihydrocoumarin phenocopies auxin action and provide a basis for further native validation in *E. crus−galli*.

## 2. Materials and Methods

### 2.1. Plant Materials and Growth Conditions

The *E. crus−galli* population used in this study was the same as that described in our previous work [32]. *E. crus−galli* seeds were treated with 98% sulfuric acid for 4–5 min to remove glumes, thoroughly rinsed with distilled water, and plump seeds were subsequently selected. Seeds of wild−type rice (WT, *Oryza sativa* cv. Zhonghua 11) and transgenic rice lines were soaked at 28 °C for 2 days, rinsed with distilled water, and subsequently germinated in an incubator maintained at 28 °C. Uniformly germinated rice seeds with visible radicle emergence were transferred to hydroponic boxes. Both rice and *E. crus−galli* were cultivated in a light incubator with a 14 h light/10 h dark photoperiod at 28 °C (light)/26 °C (dark). For each genotype–treatment combination, three replicate containers were established per independent experiment, with eight seedlings per container, and the experiment was independently repeated three times. For E. crus−galli, each replicate container consisted of a plastic cup (milk−tea cup) containing agar−solidified medium; for rice, each replicate container was a hydroponic container.

### 2.2. Bioinformatics Analysis of EcIAA9

First, based on the transcriptomic data from our previous study, *EcIAA9* was identified as the most significantly differentially expressed *AUX/IAA* gene in *E. crus−galli* under dihydrocoumarin treatment (Supplementary Figure S1A). Homologous amino acid sequences of *EcIAA9* from various species were retrieved from the NCBI database (https://www.ncbi.nlm.nih.gov). All sequences were compiled into a FASTA file, which served as the input for subsequent analyses. Multiple sequence alignment was performed using ClustalW or MUSCLE in MEGA 11, and the aligned results were saved in FASTA format. Subsequently, a phylogenetic tree was constructed using the Neighbor−Joining (NJ) method in MEGA 11 based on the aligned sequence file [46].

### 2.3. Subcellular Localization Assay

To investigate the subcellular localization of *EcIAA9*, the entire CDS of *EcIAA9* was amplified using primers specific for this gene and cloned into the pCAMBIA1300−EGFP vector. The recombinant construct and the empty vector (p2300−35S−H2B−mCherry) were separately transformed into *Agrobacterium tumefaciens* strain GV3101 (pSoup) and infiltrated into the leaves of 3− to 4−week−old *Nicotiana benthamiana* plants. After 2 days of cultivation, the EGFP fluorescence signals were observed under a laser scanning confocal microscope with excitation at 488 nm. All primers used in this study are listed in Supplementary Table S1.

### 2.4. Effect of Dihydrocoumarin on EcIAA9 Relative Expression in E. crus−galli

Uniform and plump *E. crus−galli* seeds (prepared as described above) were cultivated on agar medium following our previously reported work [31]. Two days after sowing, dihydrocoumarin solutions (Macklin, 99.68%, CAS: 119−84−6) at 10 mg/L and 20 mg/L were added separately, with the liquid level maintained at approximately 1 cm above the agar surface. Samples were then collected at 4 h, 12 h, 24 h, and 48 h post−treatment. Total RNA was extracted from the collected samples using the Vazyme RNA Extraction Kit, followed by reverse transcription into cDNA. Specific primers corresponding to the *EcIAA9* CDS were designed and used for quantitative reverse transcription PCR (qRT−PCR). The expression level was normalized to the reference gene *Actin*. Each assay included three biological replicates and three technical replicates. Relative expression levels were calculated using the 2^−ΔΔCt^ method, and statistical analyses were performed using one−way analysis of variance (ANOVA).

### 2.5. Construction of EcIAA9−Overexpression Vector and Its Homologous Genes Knockout Vectors in Rice

The full−length 516 bp fragment of *EcIAA9* was amplified via PCR using primers. The purified amplicon was cloned into pCAMBIA−1300−EGFP under the CaMV 35S promoter using BamHI/SacI restriction sites, and the resulting overexpression construct was confirmed by Sanger sequencing. For CRISPR/Cas9−mediated editing of *OsIAA9* in rice (*Oryza sativa* cv. Zhonghua 11), sgRNAs were designed using CRISPR−GE (http://skl.scau.edu.cn/) [47] targeting the first exon of *OsIAA9* (Supplementary Figure S2A). sgRNA expression cassettes were assembled into the P35S−Cas9 vector via Golden Gate cloning, followed by sequencing validation. The two recombinant vectors were introduced into *A. tumefaciens* EHA105 by freeze–thaw transformation. Agrobacterium−mediated rice transformation was performed as described previously, using embryogenic calli derived from mature seeds of Zhonghua 11 [48].

### 2.6. Effects of Dihydrocoumarin and IAA on the Growth of E. crus−galli and Rice

An agar−based bioassay was performed to evaluate the effects of dihydrocoumarin and IAA(Solarbio, 98%, CAS: 87−51−4) on the growth of *E. crus−galli*, whereas hydroponic experiments using Hoagland nutrient solution were conducted to investigate their effects on WT and transgenic rice lines. IAA powder was first dissolved in ethanol and subsequently diluted into the aqueous solution prior to treatment, yielding a final solvent concentration of 1% ethanol (*v*/*v*). Solvent−matched mock controls received the same volume of ethanol. Two days after sowing, *E. crus−galli* seedlings were exposed to serially diluted dihydrocoumarin solutions (1, 5, 10, 20 and 25 mg/L), and IAA solutions (0.05, 0.5, 5, 10, and 50 mg/L), and samples without any treatment were used as the control (CK), with three biological replicates per concentration. After 5 days of incubation in a growth chamber, root and shoot lengths were measured, and inhibition rates for root and shoot growth were calculated relative to the untreated control. For rice seedlings, plants were pre−cultured for 7 days in hydroponic boxes before dihydrocoumarin (25 mg/L) was supplied to characterize the responses of transgenic rice lines to dihydrocoumarin. To investigate the functional relationship between IAA and dihydrocoumarin in WT and transgenic rice lines, four treatment groups were applied immediately after seed radicle emergence: dihydrocoumarin alone (2 mg/L), IAA alone (1 mg/L), combined dihydrocoumarin (2 mg/L) plus IAA (1 mg/L), and water−treated control. Five days after treatment, root length was measured, and root growth inhibition rates were determined relative to the untreated control.

### 2.7. Yeast Two−Hybrid Assay

Based on the core components of the auxin signaling pathway and our previous transcriptomic data, four candidate genes (*scaffold41.26*, *scaffold149.50*, *scaffold17.633* and *scaffold5.205*) were selected from a pool of seven for the identification and validation of proteins interacting with *EcIAA9* (Supplementary Figure S1B). The CDSs of *scaffold41.26*, *scaffold149.50*, *scaffold17.633* and *scaffold5.205* were obtained as described previously. Yeast two−hybrid (Y2H) assays were conducted using the Matchmaker™ Gold Yeast Two−Hybrid System (Clontech, Mountain View, CA 94043, USA) with yeast strain Y2HGold. The bait vector pGBKT7 (BD, TRP1) and prey vector pGADT7 (AD, LEU2) were used for fusion protein expression. All constructs were confirmed by sequencing. Yeast competent cells were prepared using the LiAc method. Co−transformations of pGBKT7−*EcIAA9* with prey plasmids (e.g., pGADT7−scaffold41.26, scaffold149.50, scaffold17.633 and scaffold5.205) were performed, and cells were spread on SD/−Leu/−Trp (DDO) plates. After 3–5 days at 30 °C, colonies were transferred to SD/−Leu/−Trp/−His/−Ade (QDO), and colony growth on SD/−Leu/−Trp/−His/−Ade were scored as positive interactions. Positive control (pGBKT7−53 + pGADT7−T) and blank control (pGBKT7 + pGADT7) were included in all experiments. All interactions were verified in at least three independent trials.

### 2.8. Luciferase (LUC) Complementation Imaging Assay

*EcIAA9* was cloned into pCAMBIA1300−CLUC, while *scaffold41.26*, *scaffold149.50*, *scaffold17.633* and *scaffold5.205* were inserted into pCAMBIA1300−NLUC. All constructs were sequence−verified for correct in−frame fusions. Recombinant plasmids were transformed into *A. tumefaciens* GV3101 (pSoup) via freeze–thaw method. Overnight cultures (LB with 50 μg/mL kanamycin, 25 μg/mL rifampicin) were resuspended to OD_600_ = 0.8 in infiltration buffer (10 mM MgCl2, 10 mM MES pH = 5.6, 150 μM acetosyringone) and incubated for 2 h at 25 °C. Equal volumes of complementary agrobacterial suspensions were co−injected into 4−week−old *N. benthamiana* leaves. Plants were grown at 25 °C (16 h light/8 h dark) for 48 h. Leaves were treated with 1 mM luciferin, incubated in the dark for 5 min, and imaged using a plant in vivo imaging system. Each assay included three biological replicates.

### 2.9. Generation and Genotyping of OsARF7−*KO* Lines

The *OsARF7*−KO rice (T_2_ generation seeds, No. BG110011G01, Batch M1109, Date 9 November 2023) was purchased from Biogle Gene Company (Hangzhou, China). In the experiment, sequencing was performed using the verification primers provided by this company, and sequence alignment was carried out with the SnapGene software (4.2.4). Genotyping verification confirmed that there was a 2 bp insertion in the *OsARF7* gene (Supplementary Figure S2B), and the mutant was a homozygous mutant.

### 2.10. Statistical Analyses

The experiments were performed with at least three independent biological replicates. One−way analysis of variance (ANOVA) followed by Tukey’s multiple comparison test was performed for multi−group statistical comparisons, and the Mann–Whitney U test was used for−comparisons between two groups. Data visualization and figure preparation were carried out using GraphPad Prism (version 8.0) and Adobe Illustrator, respectively. Sequence alignment and genotyping validation were performed using SnapGene (version 6.0). All test data are expressed as the mean ± standard deviation (SD), and a significance level of *p* < 0.05 was used to indicate statistical significance.

## 3. Result

### 3.1. Effects of Dihydrocoumarin and IAA on E. crus−galli

**Figure 1:**
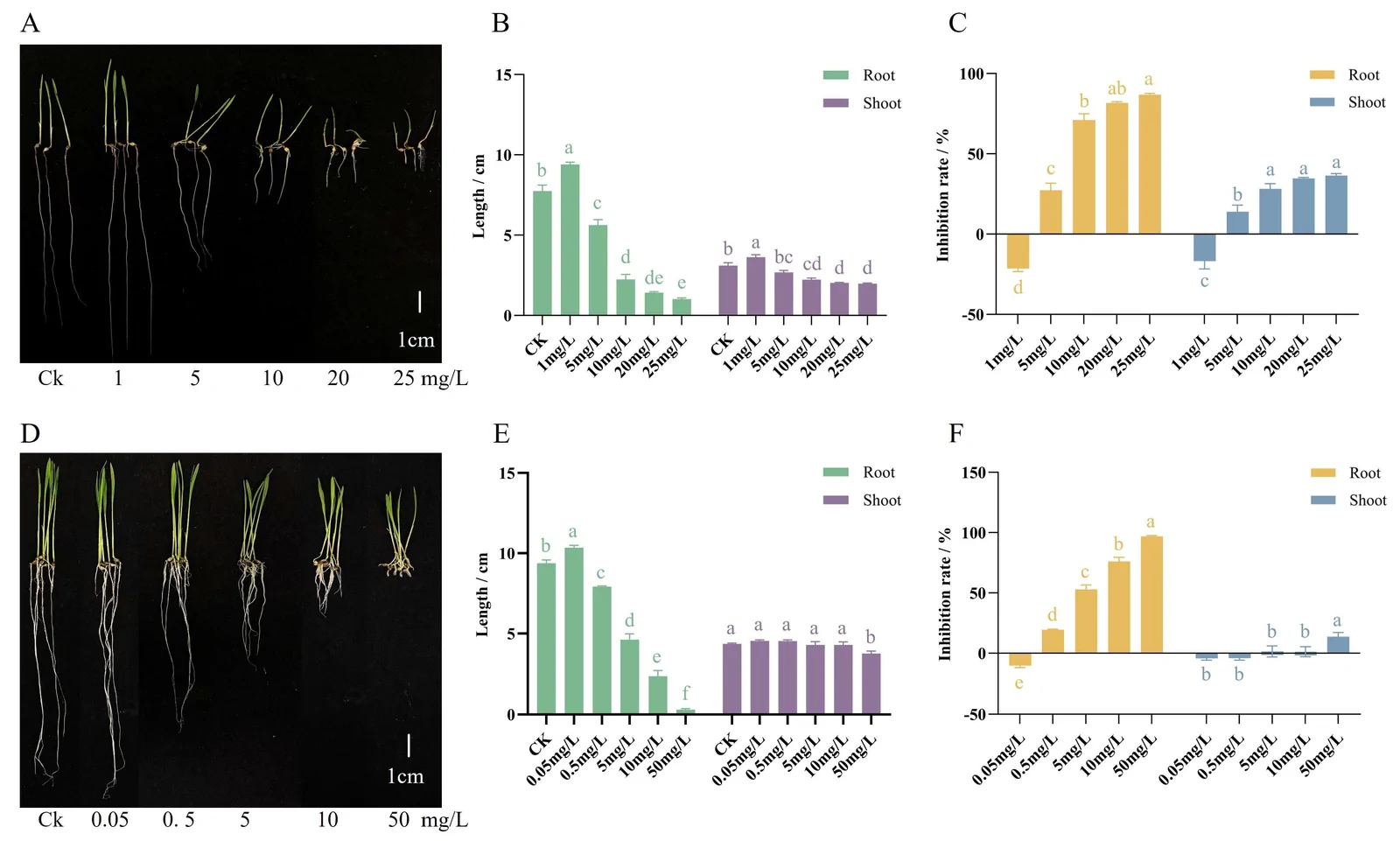
Growth responses of *Echinochloa crus−galli* seedlings to dihydrocoumarin and IAA treatments. *E. crus−galli* seedlings were treated with a range of dihydrocoumarin concentrations, IAA, and without any treatment were used as the control (CK). (**A**) Representative seedling phenotypes under dihydrocoumarin treatment. (**B**) Primary root and shoot length (cm) under dihydrocoumarin treatment. (**C**) Root and shoot growth inhibition rates under dihydrocoumarin treatment. (**D**) Representative seedling phenotypes under IAA treatment. (**E**) Primary root and shoot length (cm) under IAA treatment. (**F**) Root and shoot growth inhibition rates under IAA treatment. Inhibition rates were calculated relative to the untreated CK control. All quantitative data are shown as mean ± SD of biological replicates. Different lowercase letters indicate significant differences among treatments, one way ANOVA followed by Tukey’s multiple comparison test (*p* < 0.05).

Dihydrocoumarin affected *E. crus−galli* seedling growth in a manner dependent on concentration, stimulating elongation at low concentrations while suppressing both roots and shoots at high concentrations (Figure 1A,B). Two days after sowing, the seedlings were subjected to the treatments, and phenotypic observations were conducted five days later. At 1 mg/L, dihydrocoumarin significantly promoted root elongation, with root length increased from 7.74 cm (CK) to 9.40 cm, representing a 21.38% increase relative to the untreated control (Figure 1C). By contrast, higher concentrations (5, 10, 20, and 25 mg/L) all significantly suppressed root growth, and the strongest inhibitory effect was observed at 25 mg/L, which reduced root length by 86.80%. Shoot growth exhibited a consistent response pattern to dihydrocoumarin: at 1 mg/L, dihydrocoumarin stimulated shoot elongation, with shoot length increased from 3.11 cm (CK) to 3.63 cm, representing a 16.79% increase. Whereas concentrations of 10–25 mg/L caused significant shoot inhibition, with shoot length decreased from 2.68 cm to 1.98 cm, and the maximum reduction of 36.43% was detected at 25 mg/L (Figure 1B). These results illustrated that dihydrocoumarin exerted particularly prominent inhibitory effects on root growth.

Similarly, IAA also displayed a typical biphasic dose–response on *E. crus−galli* seedling development: low doses of IAA favored seedling growth, whereas elevated concentrations produced obvious inhibitory impacts on root performance (Figure 1D,E). Low IAA concentrations of 0.05 mg/L significantly promoted root elongation, with root length increased from 9.38 cm to 10.35 cm, representing a 10.30% increase relative to the control group. (Figure 1F). Conversely, root growth was sharply restricted when IAA concentration further increased. The most severe growth suppression was observed at 50 mg/L IAA, leading to a 96.90% reduction in root length compared with untreated seedlings. By contrast, shoot growth was far less sensitive to IAA treatment; significant shoot growth inhibition was only detected at the highest concentration of 50 mg/L. Consistent with the phenotypic pattern triggered by dihydrocoumarin, roots exhibited much stronger sensitivity than shoots. Therefore, subsequent experiments focused on root phenotypes to further dissect the phytotoxic action of dihydrocoumarin that mimics auxin.

### 3.2. Sequence Analysis of EcIAA9

**Figure 2:**
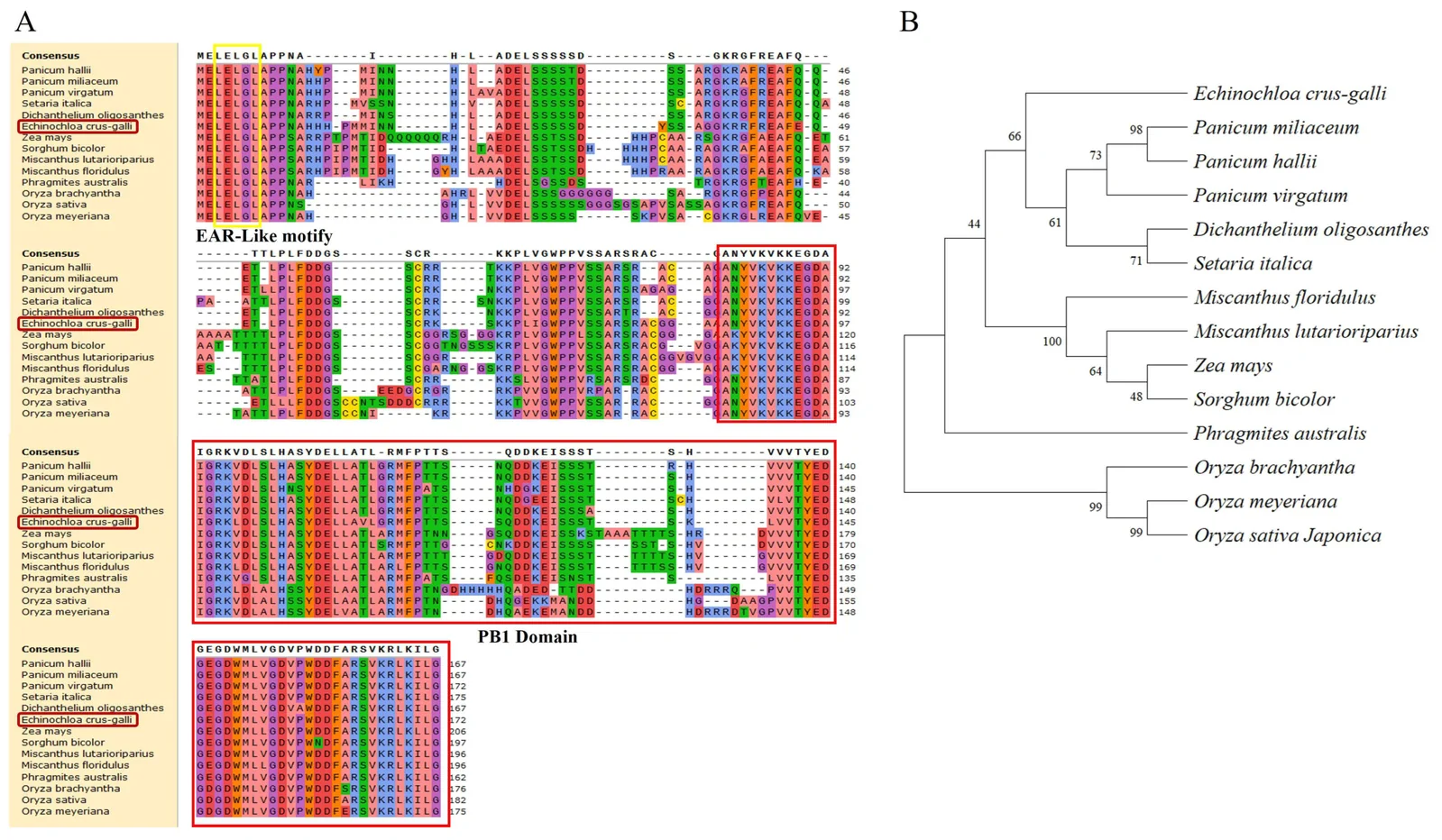
Sequence conservation and phylogenetic analysis of *EcIAA9* across plant species. (**A**) Multiple sequence alignment of Aux/IAA9 amino acid sequences shown as a matrix plot with colour coding: the EAR−like motif (yellow box) and PBI domain (red box) are marked in the alignment. (**B**) Neighbour−joining phylogenetic tree constructed based on Aux/IAA9 amino acid sequences. Bootstrap values (≥48%) are shown at branch nodes.

Transcriptomic analysis from our previous work demonstrated that dihydrocoumarin treatment markedly affects auxin signal transduction in *E. crus−galli*. *EcIAA9*, an Aux/IAA family gene, was identified as the most significantly differentially expressed gene in this pathway. Accordingly, we further characterized the sequence and structural features of *EcIAA9*. The CDS sequence of the *EcIAA9* gene was amplified via RT−PCR using *E. crus−galli* seedlings from the same population reported in our previous study [3]. The 516 bp CDS encodes a protein of 171 amino acids with a molecular weight of 18.64 kDa. The results of multiple sequence alignment showed that there were numerous conserved regions in the amino acid sequences of *EcIAA9* across different species. These conserved regions correspond to functional domains or key active sites of the *EcIAA9* protein, including the EAR−like repressor motif and the PB1 domain (Figure 2A), suggesting their functional importance throughout evolution. The Neighbor−Joining (NJ) phylogenetic tree (Figure 2B) showed two major clades. In the first, *E. crus−galli* clustered with *Panicum* spp., *Setaria italica*, and *Dichanthelium oligosanthes* (bootstrap support 66–98%). The second clade contained *Miscanthus* spp., *Zea mays*, and *Sorghum bicolor* (bootstrap 48–100%). *Oryza* spp. and *Phragmites australis* formed separate clades, with bootstrap support of 99% within the *Oryza* genus. Given the conserved domain architecture of *EcIAA9*, we next investigated its transcriptional response to dihydrocoumarin and its subcellular localization.

### 3.3. Dihydrocoumarin Upregulates EcIAA9 Expression in E. crus−galli

**Figure 3:**
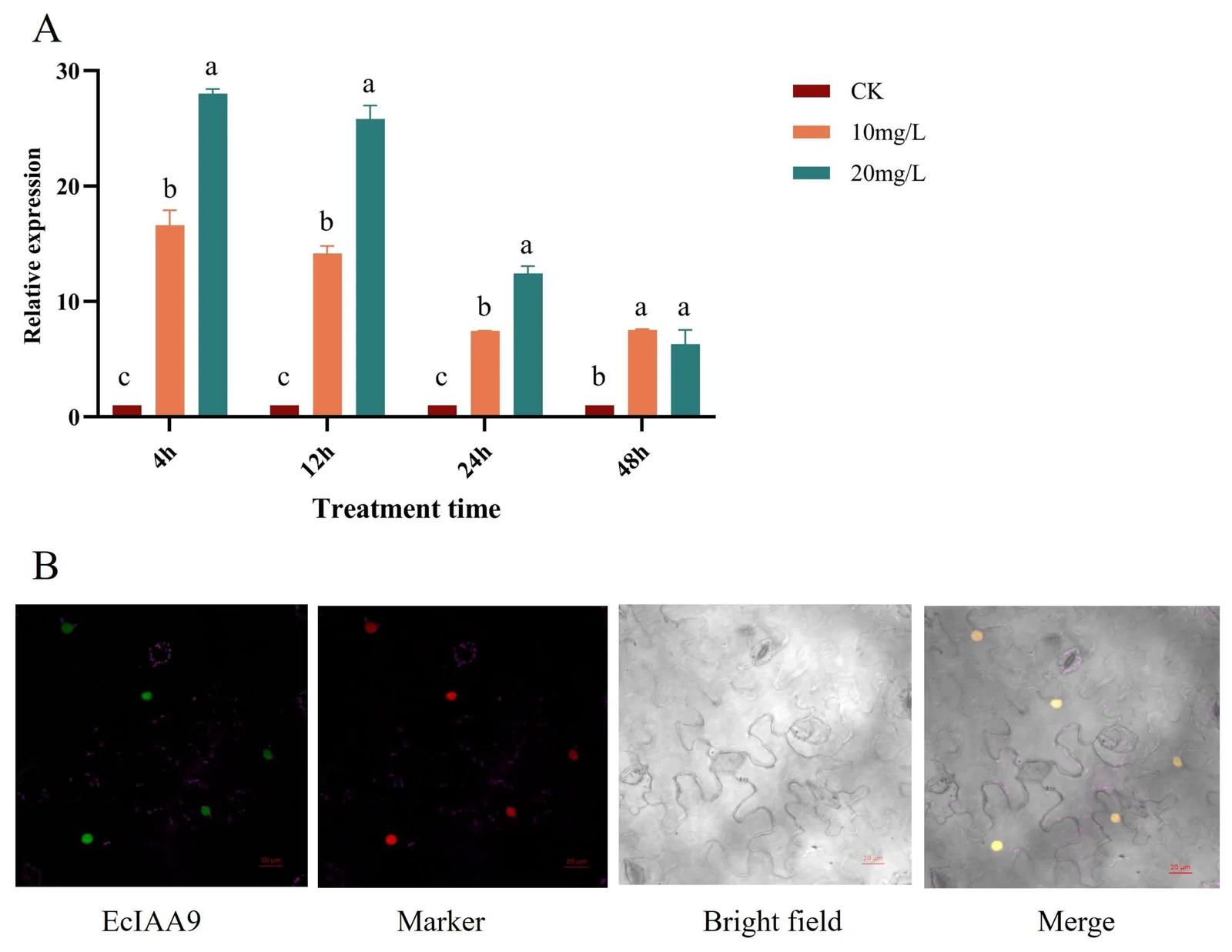
Transcript abundance and subcellular localization of *EcIAA9*. (**A**) Relative expression levels of *EcIAA9* in *E. crus−galli* seedlings treated with 10 mg/L and 20 mg/L dihydrocoumarin for 4, 12, 24, and 48 h. CK corresponds to the untreated control. Data are means ± SD, different lowercase letters indicate significant differences determined by one−way ANOVA followed by Tukey’s multiple comparison test (*p* < 0.05). (**B**) Subcellular localization of the *EcIAA9*−EGFP fusion protein transiently expressed in *Nicotiana benthamiana* leafs epidermal cells. From left to right: *EcIAA9*−EGFP fluorescence signal, nuclear marker signal, bright field image, and merged image. Scale bar = 100 μm.

RT−qPCR was used to assess *EcIAA9* expression over time after treatment with 10 and 20 mg/L dihydrocoumarin. The results showed that *EcIAA9* expression in *E. crus−galli* increased with dihydrocoumarin concentration but decreased with treatment time (Figure 3A). After 4, 12, and 24 h of treatment, *EcIAA9* expression was significantly higher in the 20 mg/L group (28.00, 25.82, 12.43) than in the 10 mg/L group (16.61, 14.17, 7.46) and the control (all *p* < 0.05). At 48 h after treatment, the two concentration groups showed similar expression levels (7.54, 6.32, respectively), with no significant difference between them, but both were significantly higher than CK. Thus, dihydrocoumarin upregulates *EcIAA9* expression, and this induction diminishes with treatment time. Given the conserved domain architecture of *EcIAA9*, we next investigated its transcriptional response to dihydrocoumarin and its subcellular localization.

### 3.4. EcIAA9 Localizes to the Nucleus

In this study, the *EcIAA9* protein was fused to the C−terminus of p1300−EGFP for heterologous expression in *N. benthamiana* leaves, with empty vector serving as a control. Following cultivation under a 12 h light/12 h dark photoperiod, confocal fluorescence microscopy showed that the IAA9−EGFP fusion protein localized to the nucleus (Figure 3B), whereas no obvious fluorescence signal was detected in empty vector control leaves. To further explore the biological function of *EcIAA9* in dihydrocoumarin−triggered root inhibition, we generated heterologous transgenic rice lines for genetic phenotypic assays.

### 3.5. Effects of Dihydrocoumarin on Root Growth in EcIAA9−OE and OsIAA9−KO Rice

**Figure 4:**
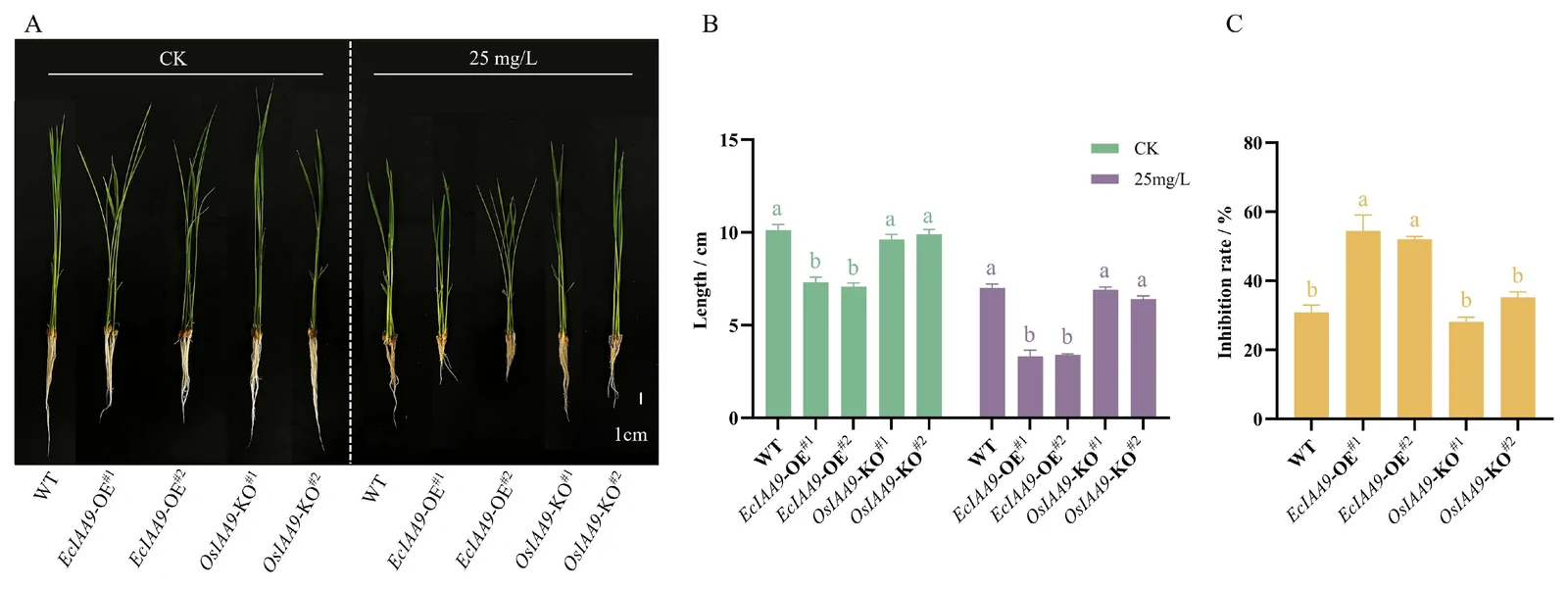
Effects of dihydrocoumarin on root growth of rice seedlings with different *EcIAA9* lines. (**A**) Phenotypic characteristics of wild−type (WT, ZH11), *EcIAA9* overexpressing (*EcIAA9*−OE), and *OsIAA9* knockout (*OsIAA9*−KO) rice seedlings under control (CK) and 25 mg/L dihydrocoumarin conditions. Rice seedlings were pre−cultured in hydroponic boxes for 5 d after germination, then subjected to treatments, and phenotypes were recorded after 5 d of treatment. Scale bar = 1 cm. (**B**) Primary root length of different rice genotypes under control and 25 mg/L dihydrocoumarin treatments. (**C**) Root growth inhibition rates of different rice genotypes induced by 25 mg/L dihydrocoumarin. All data are presented as means ± SD. Different lowercase letters indicate significant differences among genotypes based on one−way ANOVA followed by Tukey’s multiple comparison test (*p* < 0.05).

To investigate the role of *EcIAA9* in response to dihydrocoumarin, we phenotypically analyzed *EcIAA9*−OE and *OsIAA9*−KO rice lines. Two independent CRISPR/Cas9 knockout lines were generated by targeting the *OsIAA9* gene. In *EcIAA9*−OE lines (their overexpression levels were shown in Supplementary Figure S3), root lengths of *EcIAA9*−OE^#1^ and *EcIAA9*−OE^#2^ were 7.32 cm and 7.08 cm, respectively, which were significantly shorter than that of WT (10.12 cm) under untreated control conditions (Figure 4A). By contrast, the root lengths of *OsIAA9*−KO^#1^ and *OsIAA9*−KO^#2^ (9.62 cm and 9.90 cm) showed no significant difference relative to WT. Seven days after sowing, the seedlings were subjected to the treatments, and phenotypic observations were conducted five days later. Under treatment with 25 mg/L dihydrocoumarin, root elongation was suppressed in all rice genotypes (Figure 4A); nevertheless, the overall root length pattern among different genotypes remained consistent with that observed under the untreated control. The root length inhibition rate in WT was approximately 30.81% under dihydrocoumarin treatment (Figure 4B). In *EcIAA9*−OE^#1^ and *EcIAA9*−OE^#2^, the inhibition rates were 54.44% and 52.00%, respectively, which were significantly higher than that of WT. In contrast, the root length inhibition rates in *OsIAA9*−KO^#1^ and *OsIAA9*−KO^#2^ were both below 38.38%, with no significant difference compared with WT. Dihydrocoumarin might exert its phytotoxic activity through an IAA9−dependent pathway. At a later developmental stage (7−day−old) under 25 mg/L treatment, the lack of a significant difference between the KO lines and WT may be attributed to the influence of other developmental regulators or compensatory mechanisms on the physiological response. These findings suggested that *IAA9* functioned as a positive regulator may act as a core component for the suppression of root elongation induced by dihydrocoumarin, although the underlying mechanism may operate at the transcriptional and/or posttranslational levels, a possibility that remains to be elucidated.

### 3.6. Identification and Verification of Proteins Interacting with EcIAA9

**Figure 5:**
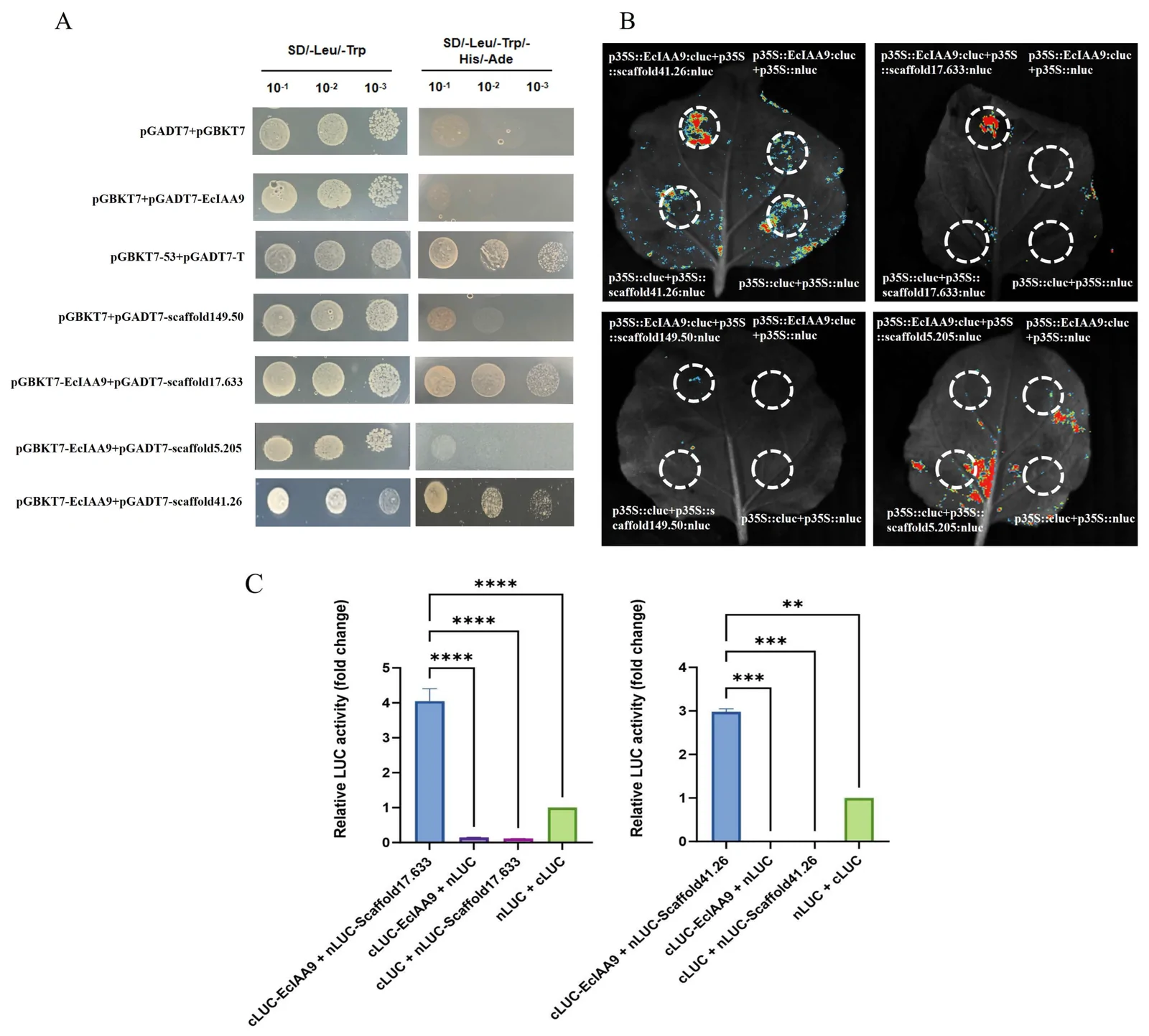
**Verification of the interaction between *EcIAA9* and four candidate proteins.** (**A**) Yeast two−hybrid (Y2H) assays for *EcIAA9* and four candidate proteins. Y2H results confirmed physical interactions between *EcIAA9* and scaffold17.633, as well as scaffold41.26. Positive control: pGBKT7−53 + pGADT7−T; negative control: pGBKT7 + pGADT7. (**B**) Luciferase (LUC) complementation imaging assay validating protein interactions in *Nicotiana benthamiana* leaves. *EcIAA9* was fused to the C−terminal fragment of luciferase (*EcIAA9*−cLUC), and each candidate protein was fused to the N−terminal luciferase fragment (candidate−nLUC). Luminescence signals verified the interactions of *EcIAA9* with scaffold17.633 and scaffold41.26. Negative controls included co−infiltration of nLUC + cLUC, *EcIAA9*−cLUC + nLUC, and cLUC + each candidate−nLUC. (**C**) Quantitative analysis of the luciferase (LUC) activity. The relative LUC activity (fold change) was measured to quantify the interaction strength between *EcIAA9* and Scaffold41.26 (left panel) or Scaffold17.633 (right panel). Co−expression of cLUC−*EcIAA9* with nLUC−Scaffold41.26 or nLUC−Scaffold17.633 resulted in significantly higher LUC activity compared to the negative controls (cLUC−*EcIAA9* + nLUC, cLUC + nLUC−Scaffold, and nLUC + cLUC). Data are presented as mean ± SD. Asterisks indicate statistically significant differences (**, *p* < 0.01; ***, *p* < 0.001; ****, *p* < 0.0001).

To verify the four candidate proteins that interact with *EcIAA9* within the auxin signaling pathway, we performed a Y2H assay. Assays for the self−activation activity of the decoy protein and toxicity experiments involving decoy/prey proteins demonstrated that all experimental yeast strains exhibited identical growth patterns on DDO and QDO medium plates (Figure 5A). Y2H assays were performed to verify the interaction between *EcIAA9* and candidate proteins encoded by four key scaffolds. Preliminary yeast two−hybrid assays verified the reliability of the bait and prey constructs used for interaction screening. The pGBKT7−*EcIAA9* construct exhibited no self−activation activity, as yeast cells harboring this vector showed no abnormal growth on QDO medium. In addition, neither pGBKT7−*EcIAA9* nor the four tested prey vectors (pGADT7−scaffold41.26, pGADT7−scaffold149.50, pGADT7−scaffold17.633, and pGADT7−scaffold5.205) caused detectable toxicity or growth inhibition in yeast cells. Subsequent interaction tests showed that yeast strains co−transformed with pGBKT7−*EcIAA9* together with pGADT7−scaffold41.26 or pGADT7−scaffold17.633 grew normally on QDO plates, and their growth activity decreased gradually along the concentration gradient. In contrast, no visible yeast growth was observed when pGBKT7−*EcIAA9* was co−expressed with pGADT7−scaffold149.50 or pGADT7−scaffold5.205, which was consistent with the growth status of the negative control (Figure 5A). These observations confirm that *EcIAA9* specifically interacts with scaffold41.26 and scaffold17.633 proteins, but not with scaffold149.50 or scaffold5.205 proteins. To further confirm these physical interactions in plant cells, we performed luciferase complementation imaging assays in *N. benthamiana*.

To eliminate false positive signals from Y2H screening, a luciferase complementation assay was performed to further verify the interactions between *EcIAA9* and the four candidate proteins. Results indicated that the fluorescence intensity in the NLUC−scaffold41.26 and scaffold17.633 + CLUC−*EcIAA9* groups were stronger than in the other three control groups (NLUC + CLUC, NLUC + CLUC−*EcIAA9*, and CLUC + NLUC−scaffold149.50 and scaffold5.205) (Figure 5B). Consistently, quantitative analysis of the relative LUC activity (fold change) further confirmed these observations, revealing that the LUC activity in the cLUC−*EcIAA9* + nLUC−Scaffold41.26 and cLUC−*EcIAA9* + nLUC−Scaffold17.633 groups was significantly higher (approximately 2.98−fold and 4.05−fold increase, respectively) than that of the negative controls (*p* < 0.01, *p* < 0.001, and *p* < 0.0001) (Figure 5C). Sequencing analysis further revealed that scaffold41.26 and scaffold17.633 share highly homologous gene sequences and both encode the *EcARF7* protein, confirming the physical interaction between *EcIAA9* and *EcARF7*. By contrast, the fluorescence intensity of the NLUC−scaffold149.50 + CLUC−*EcIAA9* and NLUC−scaffold5.205 + CLUC−*EcIAA9* groups was comparable to that of the control groups, indicating no interaction between *EcIAA9* and these two proteins. These results suggest that *EcARF7* (encoded by scaffold41.26 and scaffold17.633) serves as the downstream executor of *EcIAA9* in response to dihydrocoumarin signaling. To genetically assess whether *OsARF7* participates in dihydrocoumarin−mediated root growth repression, we further examined the phenotypic responses of *OsARF7*KO rice lines.

### 3.7. Effects of Dihydrocoumarin on Root Growth in OsARF7−KO Rice

**Figure 6:**
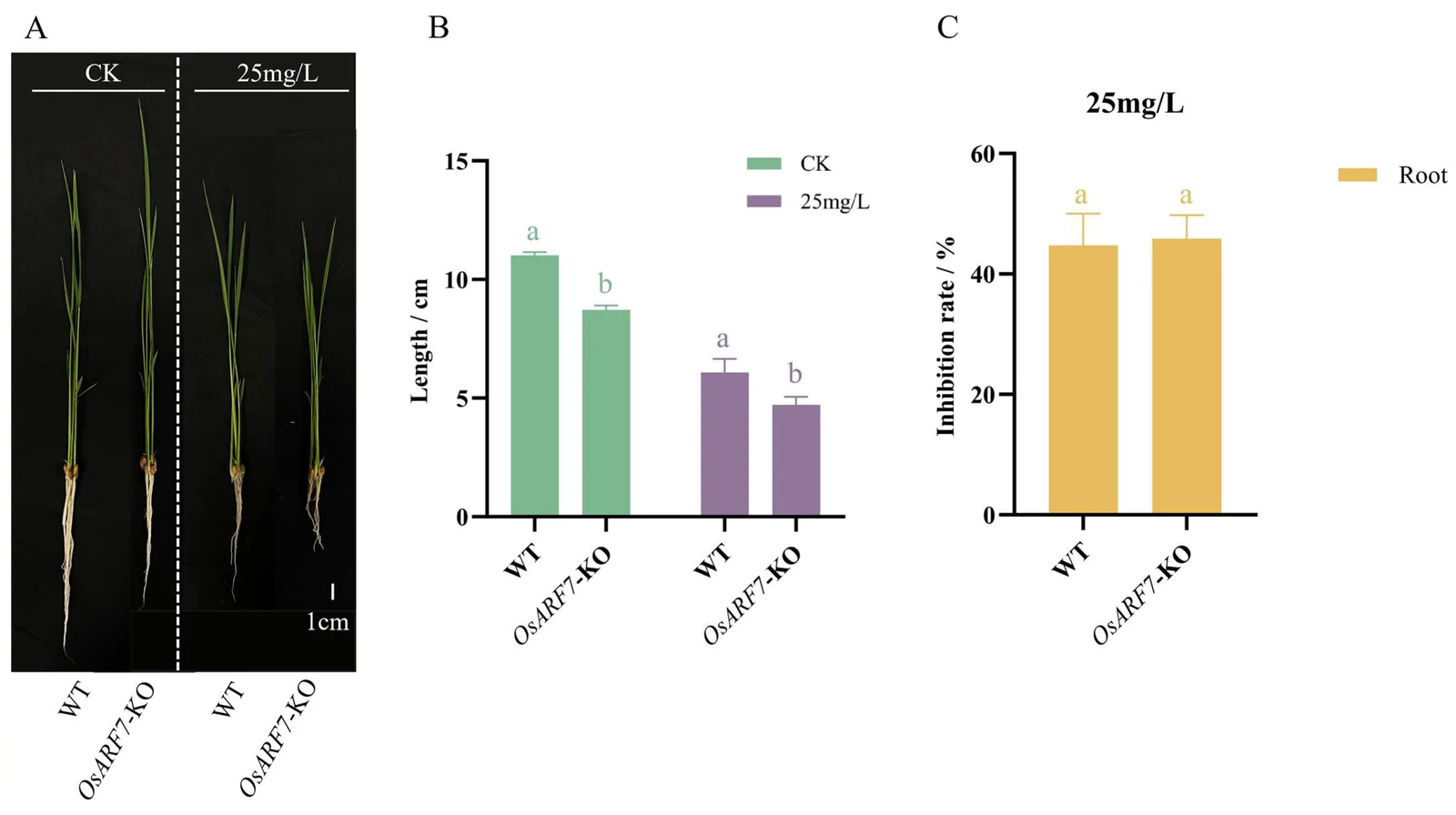
Effects of dihydrocoumarin on root growth of *OsARF7*−KO rice seedlings. (**A**) Phenotypic characteristics of wild−type (WT, ZH11) and *OsARF7*−KO rice seedlings under control and 25 mg/L dihydrocoumarin conditions. Rice seedlings were pre−cultured in hydroponic boxes for 5 d after germination, then subjected to treatments, and phenotypes were recorded after 5 d of treatment. Scale bar = 1 cm. (**B**) Primary root length of WT and *OsARF7*−KO rice under control (CK) and 25 mg/L dihydrocoumarin treatments. (**C**) Root growth inhibition rates induced by 25 mg/L dihydrocoumarin in WT and *OsARF7*−KO rice. All data are presented as means ± SD. Statistical comparisons between the two genotypes were performed using the Mann–Whitney U test, with significance set at *p* < 0.05; Different lowercase letters indicate significant differences among genotypes.

Seven days after sowing, the seedlings were subjected to the treatments, and phenotypic observations were conducted five days later. The root length of *OsARF7*−KO was 8.72 cm under untreated conditions, which was significantly shorter than that of WT (11.02 cm). At 25 mg/L dihydrocoumarin, root growth w as significantly inhibited in all tested rice lines compared with untreated controls (Figure 6A). The root length of WT was 6.08 cm after treatment, representing 44.78% inhibition relative to its untreated control; the root length of *OsARF7*−KO was 4.72 cm, with an inhibition rate of approximately 45.89%, which was similar to that of WT and not significantly different (Figure 6B). These results suggest that dihydrocoumarin−induced root growth inhibition may involve *IAA9*−dependent ARF regulation. *ARF7* may not be the sole downstream factor; other redundant ARFs may compensate for *ARF7* loss, resulting in inhibition similar to that of WT. Collectively, the present study systematically characterizes the *IAA9*−ARF signaling module that mediates dihydrocoumarin action, providing a theoretical basis for deciphering the herbicidal mechanisms of allelochemicals derived from natural coumarins.

### 3.8. Effects of Dihydrocoumarin and IAA on Root Growth in Rice

**Figure 7:**
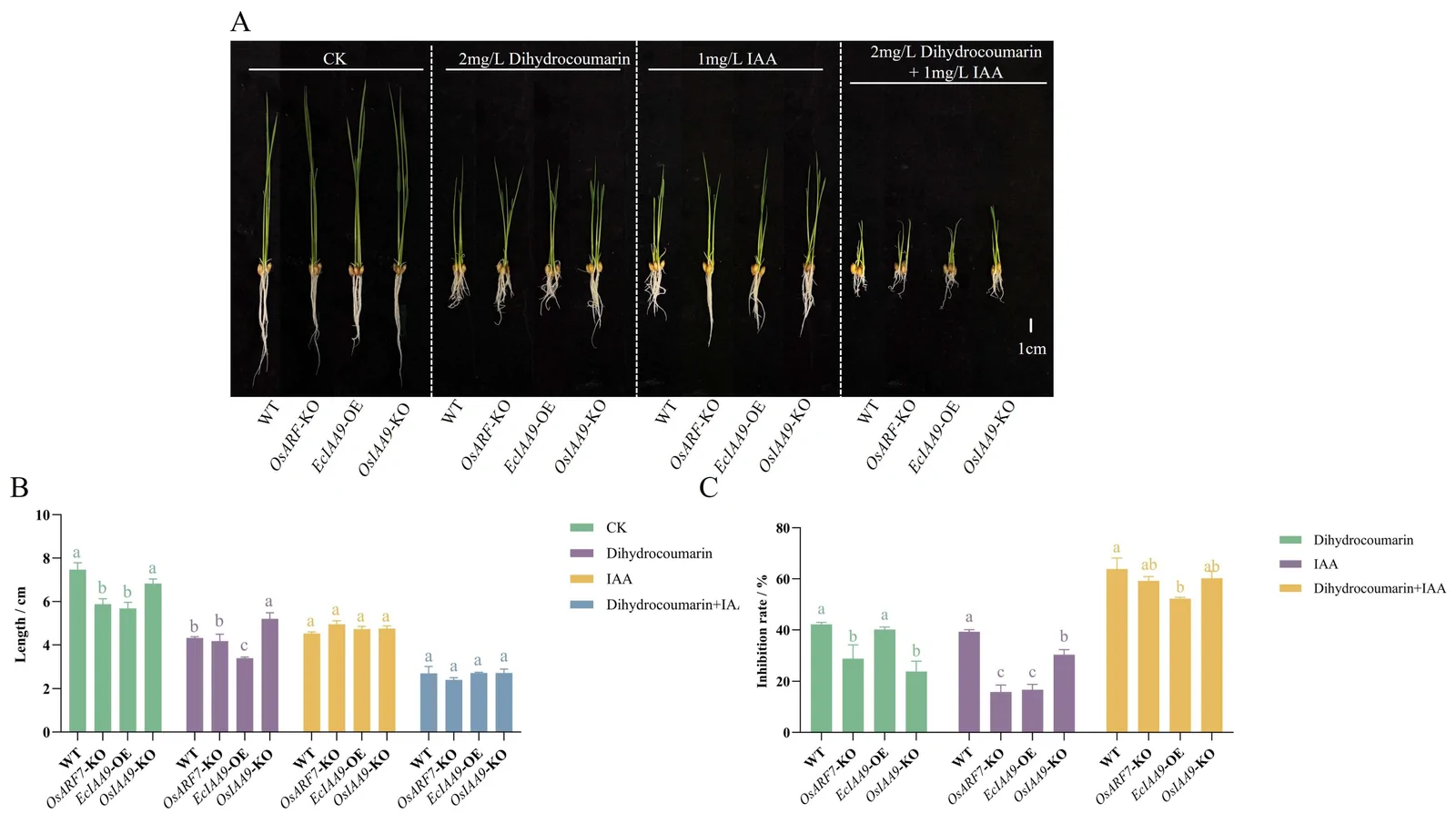
Root growth and inhibition rates of different rice genotypes under dihydrocoumarin and IAA treatments. Phenotypes, primary root length and root growth inhibition rates of rice genotypes under dihydrocoumarin and IAA treatments. Wild−type (WT, ZH11), *EcIAA9*−OE, *OsIAA9*−KO, and *OsARF7*−KO rice seedlings were hydroponically cultured and exposed to four treatments: control (CK), dihydrocoumarin, exogenous IAA, and combined dihydrocoumarin + IAA. (**A**) Representative seedling phenotypes under different treatments. Scale bar = 1 cm. (**B**) Primary root length (cm) of various rice genotypes. (**C**) Root growth inhibition rate calculated relative to the corresponding untreated CK for each genotype. All values represent mean ± SD of biological replicates. Different lowercase letters indicate significant differences according to one−way ANOVA followed by Tukey’s multiple comparison test (*p* < 0.05).

To further investigate the genetic relationship between *EcIAA9* and *OsARF7* in mediating root growth inhibition, we examined root phenotypes of *EcIAA9*−OE, *OsIAA9*−KO, and *OsARF7*−KO lines under mock, dihydrocoumarin, exogenous IAA, and combined treatments (Figure 7A). Treatments were applied immediately after seed germination, and observations were performed five days after treatment. Under mock conditions, root lengths of *EcIAA9*−OE and *OsARF7*−KO were 5.68 cm and 5.88 cm, respectively, which were significantly shorter than those of WT (7.47 cm) and *OsIAA9*−KO (6.83 cm) (Figure 7B). No significant difference was observed between *EcIAA9*−OE and *OsARF7*−KO, nor between WT and *OsIAA9*−KO. Upon 2 mg/L dihydrocoumarin treatment, WT and *EcIAA9*−OE displayed the highest inhibition rates at 42.19% and 40.18%, respectively, and were statistically equivalent (Figure 7C). In contrast, *OsIAA9*−KO exhibited the lowest inhibition rate of 23.86%, while *OsARF7*−KO showed an intermediate inhibition rate of 28.89%, with no significant difference compared with *OsIAA9*−KO. Under exogenous IAA treatment, WT exhibited the highest inhibition rate at 39.28%, significantly above all other genotypes. *OsIAA9*−KO showed an intermediate rate of 30.45%, significantly higher than those of *EcIAA9*−OE (16.71%) and *OsARF7*−KO (15.86%); no significant difference was detected between *EcIAA9*−OE and *OsARF7*−KO. When dihydrocoumarin and IAA were co−applied, all genotypes exhibited drastically shortened roots with universally high inhibition rates. *EcIAA9*−OE had the lowest rate at 52.20%, which differed significantly from WT (63.84%), whereas WT did not differ significantly from *OsIAA9*−KO (60.22%) or *OsARF7*−KO (59.21%). In addition, under this combined treatment, *EcIAA9*−OE showed no significant difference from either mutant. To further quantify the interaction between dihydrocoumarin and IAA across genotypes, Colby’s method was applied (Table S3). The combined effect was additive in WT and *EcIAA9*−OE, but synergistic in *OsIAA9*−KO and *OsARF7*−KO. Collectively, these phenotypic data suggest that both *EcIAA9* and *OsARF7* participate in the root growth inhibitory responses triggered by dihydrocoumarin and IAA. Furthermore, the contrasting sensitivity of *EcIAA9*−OE to dihydrocoumarin versus IAA indicates that this line responds differently to the two compounds. The persistent inhibitory effects observed in the mutant backgrounds suggest the possible involvement of compensatory genetic pathways.

## 4. Discussion

Natural allelochemicals represent promising eco−friendly candidates for developing bioherbicides and provide new resources for sustainable weed management [49]. Since the phytotoxicity of juglone was identified in the 1920s, numerous allelopathic compounds from crops, forest plants, and marine species have been characterized; these substances are released via root exudation, volatilization, leaching, and decomposition [15]. In recent years, research on allelopathy has shifted from identifying allelochemicals and assessing their effects on weed suppression toward elucidating phytotoxic mechanisms and corresponding biosynthetic pathways. Consistent with our previous report, the hydrogenated derivative exhibited stronger phytotoxicity against root growth of E. crus−galli than coumarin; the present work further shows that it disrupts auxin signaling via triggering the transcription of *EcIAA9*. Our findings <span suggest *EcIAA9* as a potential mediator.

From the decomposition of *M. aquaticum*, we isolated dihydrocoumarin and confirmed its phytotoxicity toward *E. crus−galli*. Functional evidence indicated that elevated *EcIAA9* appears to mediate growth inhibition induced by dihydrocoumarin, and ARF7 was characterized as its core interacting component involved in auxin signal transduction.

Dihydrocoumarin is a degradable, eco−friendly allelochemical that can be synthesized via one−step hydrogenation of coumarin, a widely distributed plant metabolite [50]. The abundance of its natural sources confers economic advantages, and its low toxicity and biosafety are well documented [51,52]. Furthermore, the strong inhibitory effects we observed on the root growth of *E. crus−galli* support its potential as a weed control agent, suggesting it may be a favorable candidate for further development. However, the documented low toxicity and natural abundance reported in the previous literature do not by themselves constitute field−level environmental safety or economic advantages. In terms of its parent compound coumarin, metabolomic and transcriptomic analyses have demonstrated that coumarin disrupts the tricarboxylic acid cycle in *Eleusine indica* [53]. Accumulating evidence has confirmed that coumarin exhibits auxin−like biological activities. Lupini revealed that the suppression of root growth by coumarin is mainly associated with altered expression of PIN2 and AUX1, key proteins involved in auxin transport [54]. Furthermore, genetic screening using *Arabidopsis* mutants indicated that root growth inhibition triggered by coumarin depends on the core neddylation enzyme NAE (NEDD8 activating enzyme), which modulates the degradation of Aux/IAA proteins [45]. Notably, phenotypic characterization of *E. crus−galli* seedlings under dihydrocoumarin and IAA treatments revealed a typical biphasic effect on root growth that depended on concentration, with low concentrations promoting root elongation while high concentrations exerted inhibitory effects (Figure 1). These parallel phenotypic responses raise the possibility that dihydrocoumarin may interfere with root development by perturbing the same regulatory pathway related to auxin. This notion is supported by the established modulation of root architecture by concentration, where appropriate auxin signaling sustains normal root development, whereas hyperactivated auxin responses disturb growth homeostasis [55,56]). Nevertheless, few studies have addressed whether structural modifications such as hydrogenation reshape its phytotoxic mechanism. Our results reveal that dihydrocoumarin, its hydrogenated derivative, exhibits stronger phytotoxicity against root growth of *E. crus−galli* than coumarin [31]. In addition, it disrupts auxin signaling via triggering the transcription of *EcIAA9*.

Our findings suggest *EcIAA9* as a potential mediator that appears to mediate the disturbance of auxin signaling induced by dihydrocoumarin. Consistent with the conserved functions of Aux/IAA family genes across plant species, accumulating evidence demonstrates that *IAA9* homologs execute versatile regulatory roles in root development in both model and crop plants. In *Arabidopsis*, *AtIAA9* is involved in adventitious root initiation through the modulation of jasmonic acid homeostasis [57]. In rice, *OsIAA9* is specifically expressed in lateral roots and root tips, where it regulates the number of crown and lateral roots and mediates root growth inhibition induced by ethylene [38,58]. Moreover, transgenic *Arabidopsis* plants overexpressing *OsIAA9* exhibit increased lateral root number and reduced gravitropic response [59].

Building on the above results, we generated *EcIAA9*−OE and *OsIAA9*−KO rice lines and examined their root phenotypes under dihydrocoumarin treatment. Under normal growth conditions, heterologous overexpression of *EcIAA9* significantly suppressed root elongation, whereas knockout of endogenous *OsIAA9* had no obvious effect on root growth. After dihydrocoumarin treatment, relative root growth inhibition was calculated against each line’s own untreated control; *OsIAA9*−KO did not show significantly stronger inhibition than WT but differed significantly from *EcIAA9*−OE (Figure 4). This may involve developmental stage, other developmental regulators, or compensatory mechanisms, and remains hypothetical. These findings suggest that *OsIAA9* may participate in mediating dihydrocoumarin−triggered root growth inhibition. Notably, although *EcIAA9* itself has root−repressive activity, *EcIAA9*−OE showed significantly higher relative inhibition than WT after dihydrocoumarin treatment, indicating that *EcIAA9* overexpression can further enhance dihydrocoumarin−induced root growth inhibition.

Aux/IAA modulate auxin signaling via dynamic interactions with ARF transcription factors, a core mechanism of this pathway [60]. At low auxin levels, Aux/IAA proteins dimerize with ARFs and repress the transcriptional activation mediated by these ARFs; at high auxin levels, Aux/IAA proteins are degraded via the TIR1/AFB pathway by the 26S proteasome, releasing ARF activity [32,60,61,62]. Given that dihydrocoumarin upregulates *EcIAA9*, we hypothesized that elevated *EcIAA9* may enhance ARF repression, thereby contributing to root growth inhibition. In support of this possibility, IAA9 homologs from rice and *Arabidopsis* have been shown to interact with specific ARFs (*OsIAA9*−ARF5; AtIAA9−ARF8), although their interaction profiles differ [57,59]. Using Y2H and luciferase complementation assays, we confirmed the physical interaction between *EcIAA9* and ARF7, suggesting that ARF7 is a potential partner of *EcIAA9* in our system. Notably, *OsARF7* has been reported to positively regulate root development [63,64], making it a plausible downstream mediator of the root inhibitory effect induced by dihydrocoumarin. To genetically test this hypothesis, we first examined the *OsARF7*−KO line under dihydrocoumarin treatment. Although the root growth inhibition rate of *OsARF7*−KO was comparable to that observed in WT (Figure 5C), no statistically significant difference was detected between the two genotypes. These results indicate that ARF7 is not the sole downstream executor of IAA9 within this regulatory pathway. We further phenotyped *EcIAA9*−OE, *OsIAA9*−KO, and *OsARF7*−KO lines under mock, dihydrocoumarin, IAA, and combined treatments. Under CK conditions, *EcIAA9*−OE and *OsARF7*−KO exhibited intrinsically shorter roots relative to WT and *OsIAA9*−KO. Under dihydrocoumarin exposure, WT and *EcIAA9*−OE showed comparable maximal inhibition, while inhibition was markedly attenuated in *OsIAA9*−KO; *OsARF7*−KO displayed intermediate responses. Upon IAA treatment, WT had the strongest root growth inhibition, *OsIAA9*−KO showed moderate sensitivity, whereas *EcIAA9*−OE and *OsARF7*−KO were less inhibited and indistinguishable. Combined treatment with dihydrocoumarin and IAA exacerbated the repression of root growth in all genotypes; *EcIAA9*−OE had the lowest inhibition rate and differed significantly from WT. Notably, *EcIAA9*−OE exhibited opposite sensitivity: susceptible to dihydrocoumarin but tolerant to IAA. This indicates that dihydrocoumarin phenocopies IAA phenotypically but functions maybe through distinct molecular routes. Whereas IAA triggers Aux/IAA degradation, dihydrocoumarin upregulates *EcIAA9* transcription, likely increasing repressive *EcIAA9* protein abundance. The severe inhibition under co−treatment may result from auxin signaling pathway overload. Overall, IAA9 may serve as a key regulatory node, but ARF7 is not the sole downstream executor; other ARF members and parallel pathways maybe provide functional compensation.

In our previous studies, we demonstrated that dihydrocoumarin exerts phytotoxic effects in *E. crus−galli*, including inducing oxidative damage, disrupting cell membrane integrity, reducing cell activity, and ultimately leading to root cell death [31]. Transcriptomic analyses revealed that dihydrocoumarin perturbs plant hormone signal transduction, with marked upregulation of *EcIAA9*. Building on these prior transcriptomic clues, the present study further dissected the auxin signaling module mediated by *EcIAA9*−*ARF7*. Evidence from genetic and protein interaction analyses indicated that the *EcIAA9*−*ARF7* regulatory module constitutes one of the key branches mediating root growth repression triggered by dihydrocoumarin, rather than the sole mechanism underlying its action. Notably, although dihydrocoumarin phenocopies the inhibition of root growth caused by IAA at the phenotypic level, our genetic assays support mechanistic divergence between these two effectors, and are consistent with a possible functional compensation among ARF family members, which remains to be tested. Our results provide a mechanistic framework for understanding the phytotoxic activity of dihydrocoumarin, illustrating the molecular complexity underlying allelochemical phytotoxicity, which integrates oxidative damage and auxin signaling perturbation. This study advances our mechanistic understanding of crosstalk between allelochemical action and auxin signaling. While dihydrocoumarin is a promising allelopathic candidate for bioherbicide development, its environmental safety and economic feasibility under agricultural field conditions require comprehensive evaluation, including non−target organism risk assessment and cost–benefit analysis. All genetic validation in this study was performed in heterologous rice, which carries multiple *IAA9/ARF7* homologs and a regulatory context distinct from E. crus−galli [65,66,67]. The *EcIAA9–ARF7* module should therefore be interpreted as a putative regulatory unit supported by heterologous evidence. Native validation via transient knockdown/overexpression of *EcIAA9* in *E. crus−galli* is required in future work to confirm its in−planta role.

## 5. Conclusions

In this study, we isolated dihydrocoumarin, an allelochemical from *M. aquaticum*, and confirmed its phytotoxicity against the noxious weed *E. crus−galli*. Dihydrocoumarin inhibited root growth in a manner dependent on concentration, showing a phenotype similar to that induced by exogenous IAA treatment. We further characterized *EcIAA9*, an Aux/IAA family gene strongly induced by dihydrocoumarin. *EcIAA9* localizes to the nucleus and physically interacts with *EcARF7*−like proteins. Genetic assays in a heterologous rice system demonstrated that *EcIAA9* contributes to root growth repression triggered by dihydrocoumarin, acting as an important branch within regulation dependent on auxin signaling rather than the sole functional pathway. Taken together, these findings provide new molecular insights into the phytotoxic mechanism of dihydrocoumarin and establish the *EcIAA9*–*EcARF7* module as a key branch in crosstalk between allelochemical action and auxin signaling.

## Data Availability

The original contributions presented in this study are included in the article/Supplementary Material. Further inquiries can be directed to the corresponding authors.

## Supplementary Materials

The following supporting information can be downloaded at: https://www.mdpi.com/article/10.3390/biology15211989/s1. Figure S1. Expression changes of Aux/IAA family genes and candidate proteins interacting with EcIAA9 in Echinochloa crus-galli following dihydrocoumarin treatment; Figure S2. Schematic of target sites and sequencing validation of OsIAA9 and OsARF7 knockouts; Figure S3. RT-qPCR validation of EcIAA9 overexpression lines; Table S1. Primers used in this study; Table S2. absolute primary-root length (cm); Table S3. Colby’s analysis of the combined effects of dihydrocoumarin and IAA on primary root growth inhibition.
