# Supplementary material for: Dihydrocoumarin Transcriptionally Activates EcIAA9 to Repress Root Growth of Echinochloa crus−galli
Source: Biology (Basel). 2026 Nov 3;15(21):1989. doi: 10.3390/biology15211989 (PMC13762044; doi:10.3390/biology15211989)
Supplement: Supplementary file 1 [file biology-15-01989-s001.zip › biology-4643874-supplementary.pdf]

Dihydrocoumarin Transcriptionally Activates *EcIAA9* to Repress Root Growth of *Echinochloa crus−galli*

Xuan Chen [1,2], Jiadi Zhang [2,3], Jiali Chen [1,2], Di Wu [1,4], Nufen Li [2,3], Shangfeng Zhou [2,4], Lifeng Wang [1,2,3,4,*] and Haona Yang [2,3,4,*]

[1] Longping Agricultural College, Hunan University, Changsha 410128, China
[2] Hunan Institute of Plant Protection, Hunan Academy of Agricultural Sciences, Changsha 410128, China
[3] College of Plant Protection, Hunan Agricultural University, Changsha 410128, China
[4] Yuelushan Laboratory, Changsha 410128, China
* Correspondence: haonayang@hunaas.cn (H.Y.); ifwang@hunaas.cn (L.W.); Tel.: +86−136−4731−5853 (H.Y.); +86−135−7484−8933 (L.W.)

Supplemental information

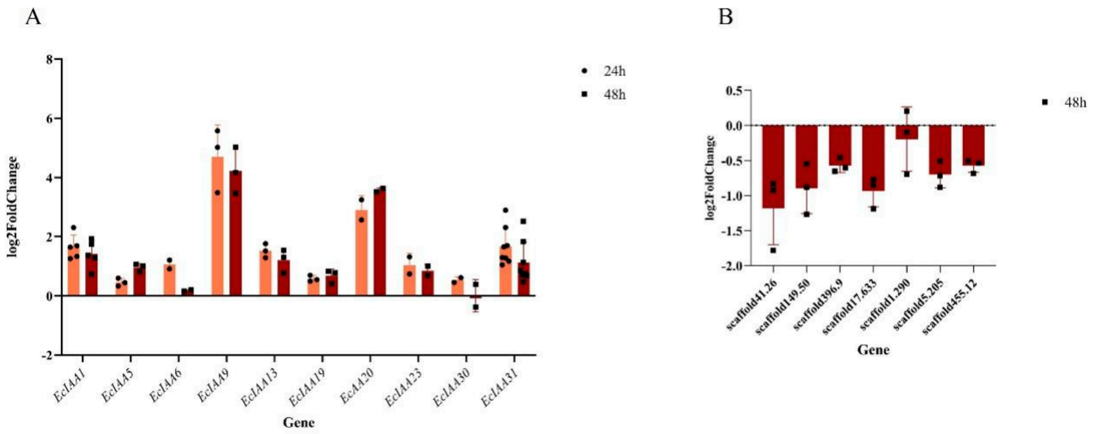

Supplementary Figure S1. Expression changes of Aux/IAA family genes and candidate proteins interacting with *EcIAA9* in *Echinochloa crus-galli* following dihydrocoumarin treatment. (A) Bar chart illustrating $\log_2$ fold-changes of individual Aux/IAA family genes at 24 h (dotted bars) and 48 h (solid bars) post dihydrocoumarin treatment. X-axis indicates gene identifiers; y-axis shows $\log_2$ fold change relative to time-matched controls. Error bars represent SD of biological replicates. (B) Bar chart showing $\log_2$ fold changes of seven candidate genes interacting with EcIAA9 at 48 h after dihydrocoumarin treatment, screened from transcriptome data for components related to auxin signaling. X-axis shows scaffold gene IDs; y-axis represents $\log_2$ fold change relative to the control after 48 h. The 24 h time point was omitted because expression variation was negligible. Four candidates (*scaffold41.26*, *scaffold149.50*, *scaffold17.633*, *scaffold5.205*) with the most prominent expression alterations were selected for subsequent protein-protein interaction assays with EcIAA9. Error bars represent SD of biological replicates.

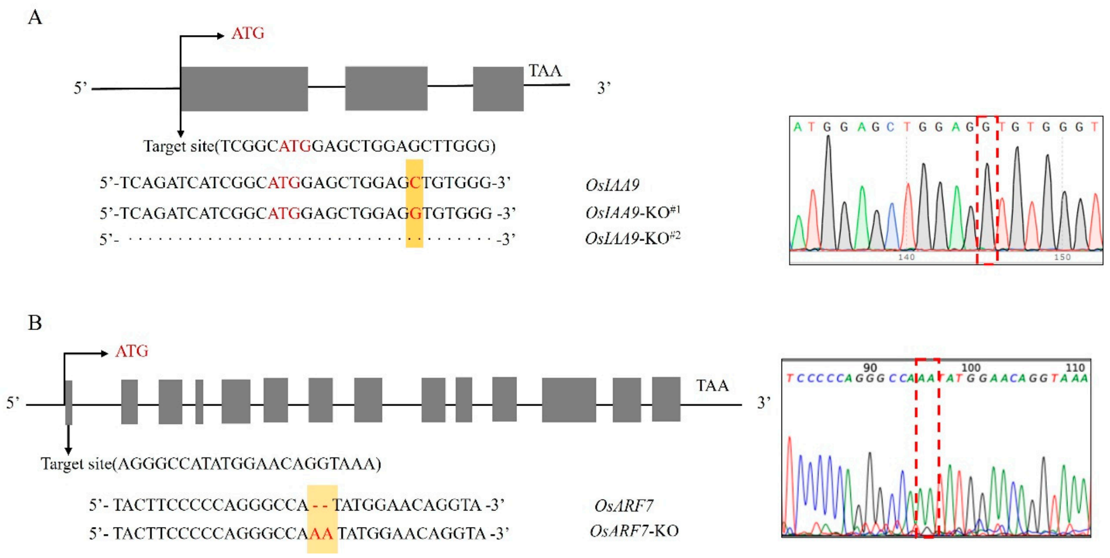

Supplementary Figure S2. Schematic of target sites and sequencing validation of OsIAA9 and OsARF7 knockouts. (A) Schematic of gRNA target sites and Sanger sequencing peak validation for *OsIAA9* knockout. (B) Schematic of gRNA target sites and Sanger sequencing peak validation for *OsARF7* knockout. Exons and introns are shown as boxes and lines, respectively. Mutation sites are shaded orange in gene structure schematics and indicated by red dashed boxes in sequencing chromatograms.

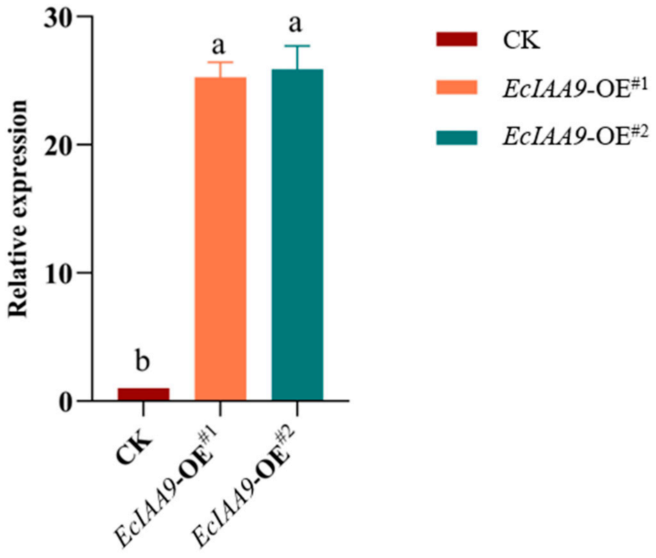

Supplementary Figure S3. RT-qPCR validation of *EcIAA9* overexpression lines. Relative *EcIAA9* transcript levels in wild–type rice (WT, *Oryza sativa* cv. Zhonghua 11) and two overexpression lines, normalized to *EcActin* and expressed as fold change relative to the WT control (set to 1.0). Both *EcIAA9-OE*$^{\#1}$ and *EcIAA9-OE*$^{\#2}$ showed 20-fold higher expression. Data: mean ± SD (*n*= 3 biological replicates). Different letters above bars denote significant differences ($P < 0.05$).

Table S1. Primers used in this study.

Purpose	Primer name	Sequence (5′→3′)
Cloning of *EcIAA9*	*EcIAA9*-F	ATGGAGCTGGAGCTCGGGCTGG
	EcIAA9-R	ATCGTGCAAAATCATCCCATGGC
Overexpression detection	*EcIAA9*-OE-F	CCGGGTACCATGGACTTCAGCGGTG
	EcIAA9-OE-R	CCGACGCGTATCGTGACCGATGTCCCCA
CRIPSR/Cas9 for OsIAA9	*EcIAA9*-KO-gRT1	ATGGGATGATGCGCGTTCGGGTTTTAGAGCTAGAAAT
	EcIAA9-KO-OsU6aT1	CCGAACGCGCATCATCCCATCGGCAGCCAAGCCAGCA
	U-F	CTCCGTTTTACCTGTGGAATCG
	GR-R	CGGAGGAAAATTCCATCCAC
	PPS-R	TTCAGAGGTCTCTACCGACTAGTCACGCGTATGGAATCGGC AGCAAA
	PGS-2	AGCGTGGGTCTCGTCAGGGTCCATCCACTCCAAGCTC
	SP-L1	GCGGTGTCATCTATGTTACTAG
	SP-R	TGCAATAACTTCGTATAGGC
Knockout detection	*EcARF7*-KO-F	CCTAAAAAAAGAGAGTAAAA
	EcARF7-KO-R	CAAAGGAAAGACATAAGATT
Yeast two-hybrid	pGBKT7-EcIAA9-F	CCGGAATTCATGGAGCTGGAGCTCGGGCTGG
	pGBKT7-EcIAA9-R	CCG GGATCCATCGTGCAAAATCATCCCATGGC
	pGADT7- scaffold149.50-F	CGGGAATTCATGGAATACTTCCCGGAGGA
	pGADT7- scaffold149.50-R	CGGGGATCCCAATATGGAGACAAATCCAGGGGCAT
	pGADT7-scaffold41.26-F	CCGGAATTCATGATCAGCGATGCTTTGTTCAGG
	pGADT7-scaffold41.26-R	CCGGGATCCGTCCTTATCGGCAGCAACT
	pGADT7-scaffold17.633-F	CCGGAATTCATGATCAGCGATGCTTTGTTCAGG
	pGADT7-scaffold17.633-R	CCGGGATCCGTCCTTATCGGCAGCAACT
	pGADT7-scaffold5.205-F	CGGCATATGATGGCGCAAGCAGCGGCAG
	pGADT7-scaffold5.205-R	CGGGGATCCAAGACCGGTCTTCAGCATGTTCTGC

Luciferase complementation imaging	EcIAA9-Cluc-F	CCGGGTACCATGGACTTCAGCGGTG
	EcIAA9-Cluc-R	CCGACGCGTACGTGACCGATGTCCCCA
	scaffold149.50-Nluc-F	CGGGGGACGAGCTCGGTACCATGGAATACTTCCCAGAGGAAGTAG
	scaffold149.50-Nluc--R	CGCGTACGAGATCTGGTCGACGTTAACGAATATGGAGACGAATCC
	scaffold41.26-Nluc-F	CCGGGTACCATGATCAGCGATGCTTTGTTCAGG
	scaffold41.26-Nluc-R	CCGACGCGTGTCCTTATCGGCAGCAACT
	scaffold17.633-Nluc-F	CCG GGTACCATGATCAGCGATGCTTTGTTCAGG
	scaffold17.633-Nluc-R	CCG ACGCGTGTCCTTATCGGCAGCAACT
	scaffold5.205-Nluc-F	CGGGGGACGAGCTCGGTACCATGGCGCAAGCAGCGGC
	scaffold5.205-Nluc-R	CGCGTACGAGATCTGGTCGACAAGACCGGTCTTCAGCATGT

Table S2. Absolute primary-root length (cm) of different plant lines under various treatments.Data are presented as mean ± SEM. Different lowercase letters within the same column indicate significant differences among different plant lines under the same treatment condition ($P < 0.05$).

	CK	25mg/L	2mg/L Dihydrocoumarin	1mg/L IAA	2mg/L Dihydrocoumarin + 1mg/L IAA
Plant line	Mean±SEM	Mean±SEM	Mean±SEM	Mean±SEM	Mean±SEM
WT	10.12±0.31a	7.00±0.22a			
EcIAA9-OE#1	7.32±0.27b	3.33±0.33b			
EcIAA9-OE#2	7.08±0.20b	3.40±0.06b			
OsIAA9-KO#1	9.62±0.27a	6.92±0.13a			
OsIAA9-KO#2	9.90±0.26a	6.42±0.16a			
WT	7.47±0.32a		4.32±0.06b	4.53±0.06a	2.70±0.32a
OsARF7-KO	5.88±0.25b		4.18±0.31b	4.95±0.16a	2.40±0.10a
EcIAA9-OE	5.68±0.28b		3.40±0.06c	4.73±0.12a	2.72±0.03a

Table S3. Colby's analysis of the combined effects of dihydrocoumarin and IAA on primary root growth inhibition in different plant lines.Data are presented as mean ± SEM. The expected response was calculated using Colby's equation:

Expected inhibition = I_DHC + I_IAA − (I_DHC × I_IAA / 100)

Difference = Observed response − Expected response. Different lowercase letters within the same row indicate significant differences between the observed and expected values ($P < 0.05$).

Inhibition rate （%）	Observed response	Expected response	Difference
WT	63.84±4.31a	64.90±0.49a	-1.0577
OsARF7-KO	59.21±1.70a	38.78±3.76b	20.4336
EcIAA9-OE	52.20±0.59a	50.18±0.85a	2.0188
OsIAA9-KO	60.22±2.72a	47.05±2.80b	13.1738
